# Landscape determinants and remote sensing of anopheline mosquito larval habitats in the western Kenya highlands

**DOI:** 10.1186/1475-2875-5-13

**Published:** 2006-02-16

**Authors:** Emmanuel Mushinzimana, Stephen Munga, Noboru Minakawa, Li Li, Chen-chieh Feng, Ling Bian, Uriel Kitron, Cindy Schmidt, Louisa Beck, Guofa Zhou, Andrew K Githeko, Guiyun Yan

**Affiliations:** 1 Climate and Human Health Research Unit, Centre for Vector Biology and Control Research, Kenya Medical Research Institute, Kenya; 2 Program in Public Health, University of California at Irvine, Irvine, CA 92697, USA; 3 National Center for Geographic Information and Analysis and Department of Geography, New York State University at Buffalo, Buffalo, NY 14260, USA; 4 Department of Pathobiology, College of Veterinary Medicine, University of Illinois, Urbana, IL 61802, USA; 5 Center for Health Applications of Aerospace Related Technologies, Ecosystem Science and Technology Branch, NASA Ames Research Center, Moffett Field, CA 94035, USA

## Abstract

**Background:**

In the past two decades the east African highlands have experienced several major malaria epidemics. Currently there is a renewed interest in exploring the possibility of anopheline larval control through environmental management or larvicide as an additional means of reducing malaria transmission in Africa. This study examined the landscape determinants of anopheline mosquito larval habitats and usefulness of remote sensing in identifying these habitats in western Kenya highlands.

**Methods:**

Panchromatic aerial photos, Ikonos and Landsat Thematic Mapper 7 satellite images were acquired for a study area in Kakamega, western Kenya. Supervised classification of land-use and land-cover and visual identification of aquatic habitats were conducted. Ground survey of all aquatic habitats was conducted in the dry and rainy seasons in 2003. All habitats positive for anopheline larvae were identified. The retrieved data from the remote sensors were compared to the ground results on aquatic habitats and land-use. The probability of finding aquatic habitats and habitats with *Anopheles *larvae were modelled based on the digital elevation model and land-use types.

**Results:**

The misclassification rate of land-cover types was 10.8% based on Ikonos imagery, 22.6% for panchromatic aerial photos and 39.2% for Landsat TM 7 imagery. The Ikonos image identified 40.6% of aquatic habitats, aerial photos identified 10.6%, and Landsate TM 7 image identified 0%. Computer models based on topographic features and land-cover information obtained from the Ikonos image yielded a misclassification rate of 20.3–22.7% for aquatic habitats, and 18.1–25.1% for anopheline-positive larval habitats.

**Conclusion:**

One-metre spatial resolution Ikonos images combined with computer modelling based on topographic land-cover features are useful tools for identification of anopheline larval habitats, and they can be used to assist to malaria vector control in western Kenya highlands.

## Background

Malaria is a major health problem in sub-Saharan Africa, where it is estimated to be responsible for over 1 million deaths every year in children younger than five and pregnant women [1]. Out of the total human population in Africa, 15% live in highlands, where there are increasing risks for epidemics [1]. Current strategies for malaria control involve treating infected individuals with anti-malarial drugs to clear the parasites, and reducing human-mosquito contact rates through vector control efforts. Anti-malarial drugs have little impact on the intensity of transmission at the community level because most drugs do not reduce the production of *Plasmodium *gametocytes, the parasite stage responsible for initiation of infection in mosquitoes [2]. Individuals who receive treatment can quickly become reinfected. Recent large field trials in Kenya demonstrated that insecticide-treated bed nets (ITN) can prevent 1 in 4 infant deaths in areas of intense perennial malaria transmission, if bed nets are used properly and re-treated with insecticide at appropriate intervals [3,4]. However, coverage and compliance are limited and emergence of insecticide-resistance genes has hindered the effectiveness of ITN programmes [5-8].

In recent years, there has been renewed interest in exploring the possibility of anopheline larval control through environmental management or larvicides as additional means of reducing malaria transmission in Africa [9-12]. Historically, eradication of the accidentally introduced African malaria mosquito *Anopheles gambiae *from north-east Brazil in the 1930s and early 1940s succeeded, through an integrated programme that relied overwhelmingly upon larval control [13]. A larval control programme successfully suppressed malaria for over 20 years around a Zambian copper mine [14] and in Dar es Salaam in Tanzania [15,16]. Source reduction through the modification of larval habitats was an important tool for malaria eradication efforts in the United States, Israel, and Italy [17]. However, the primary malaria vectors in sub-Saharan Africa, *An. gambiae *and *Anopheles arabiensis *generally utilize small temporary habitats as breeding sites [18-22], which creates difficulties for environmental management. Unfortunately, identifying these mosquito larval habitats over a large geographic area based only on field survey is time-consuming and labour intensive. Therefore, better methods for rapid and accurate determination of larval habitat distribution are critical to enable larval control using bio-insecticides or environmental modification.

Remote sensing is a powerful tool for determining the landscape features and climatic factors associated with the risk of vector-borne diseases [23-28]. For example, ecological parametres, particularly vegetation index, were found to be significantly associated with Rift Valley fever viral activity in Kenya through the National Oceanic and Atmospheric Administration's polar-orbiting meteorological satellites [29]. Climatic factors associated with malaria risks in sub-Saharan Africa were identified using climate data obtained from satellites and malaria transmission distribution maps. The malaria transmission maps were developed according to biological constraints of climate on parasite and vector development [28,30].

Remote sensing also can be used for determining factors affecting vector abundance (e.g., [31-34]) and mosquito breeding sites [35]. For instance, Beck et al. [31] analysed Landsat Thematic Mapper (TM) images of southern Chiapas, Mexico and found that transitional swamp and unmanaged pasture were the most important landscape elements for explaining vector abundance. Welch et al. [33] showed that infrared aerial photos were useful in the detection of potential oviposition sites of *Psorophora columbiae*, such as ditches, low-lying areas and tyre tracks in Texas. Roberts et al. [36] used aerial photos to determine that breeding sites located at low elevations in flooded, unmanaged pastures were the most important determinants of *Anopheles albimanus *adult abundance in southern Mexican villages. In general, previous studies demonstrate the utility of remote sensing technology in the risk assessment of vector-borne diseases and vector-population monitoring at a large spatial scale. Recently developed remote sensors of high spatial resolution may be particularly useful for determining mosquito larval habitat distribution and for assisting malaria vector control. For example, the spatial resolution is 1–4 metres for Ikonos [37], 0.61–2.44 metres for QuickBird [38], 1–4 metres for OrbView-3 [39] and 2.5–10 metres for SPOT 5 [40]. These resolutions compare favourably with 10–20 and 15–60 metres for Spot XS [40] and Landsat TM 7 [41], respectively. However, the utility of high-resolution satellite images for larval habitat identification and management have not been evaluated in African highlands where land-use pattern is highly heterogeneous and larval habitat distribution is spatially clustered [22].

The objective of this study was to assess the potential of aerial photos, Landsat TM 7 and Ikonos images for identifying larval habitats of malaria vectors and for determining other topographic features associated with anopheline larval habitats in western Kenya highlands where frequent malaria outbreaks have been reported [42-46].

## Materials and methods

### Study site

The study site is a 4 × 4 km^2 ^area in Iguhu village, Kakamega District, Western Province, Kenya (34°45" E and 0°10" N), at an elevation ranging from 1,420 m to 1,600 m above sea level. The study area contains about 2,500 households and includes a human population of about 11,000. The 1960–1999 average annual rainfall was 1,977 mm, with the long rainy season from April through June and the short rainy season from October to November. The annual mean minimum/ maximum temperature is 13.8/28.0°C, with the hottest months in January-February and the coolest months in July-August. The study area transects the Yala River valley and includes a mosaic of land-use types. Distinct landscape features in the western Kenya highlands are numerous valleys and basin-like depressions in a plateau and dramatic land-use changes such as deforestation and cultivation of natural swamps for farming. The hillside is mostly dotted with maize plantations, patches of tea (*Camellia sinensis*). Several swamps are located along the Yala River valley. A natural forest, constituting about 15% of the total area, covers the east side of the study area.

### Anopheline larval habitat distribution data acquisition

Ground surveys were conducted in February (dry season) and May (rainy season), 2003 to obtain data on all aquatic habitats and anopheline larval habitat distribution in the study area. Potential aquatic habitats included footprints and other depressions, drainage ditches, stream edges, cultivated swamps, natural swamps, pools and puddles. Occurrence of anopheline larvae in each habitat was determined using a standard dipper (350 ml). Twenty dipper collections were made in large (>0.5 m^2 ^surface area) habitats but fewer were made in small habitats. In this study, anopheline larvae were not identified to species. Minakawa et al. [47] reported species composition of anophelines in the study area as 80.5% belonging to the *An. gambiae *complex and 14.9% *Anopheles funestus*. The coordinates of each habitat were recorded using the global positioning system (GPS) in differential mode [48]. Each habitat was characterized by size (width, length, and depth), and land-cover type (forest, cultivated swamp, natural swamp, farm, pasture, shrubs, and tea plantations) as described by Minakawa et al. [47].

### Remote sensing data acquisition

Images of the study area were obtained from three remote sensors: panchromatic aerial photos, Landsat TM 7, and Ikonos satellites. Ideally, remote sensing images should be taken in the same seasons as the mosquito larval samplings; however, suitable satellite images of the study site were not available for the period of the field samplings. Using satellite images taken at different times than the larval samplings helps address the general utility of satellite images for identifying land-cover types and larval habitats across different seasons and years. Panchromatic aerial photographs were taken in February 2002 (dry season) with a ground scale of 1:10,000. The aerial photographs were scanned and geo-rectified based on 250 ground control points with GPS coordinates collected from differential GPS units. These photos were assembled into a mosaic using ERDAS Imagine software [49]. A Landsat TM 7 was taken in February 2001. A subset of the scene for the 4 × 4 km^2 ^study area was extracted from the image. A multispectral (blue, green, red, and infrared) Ikonos image [37] was taken in April 2002 (the end of dry season), with one-metre ground resolution. The image was geometrically and radiometrically corrected to account for topographic distortions and atmospheric effects [50].

### Digital elevation model (DEM) and retrieval of topographic parametres

A digital elevation model of the study area was constructed based on a contour map of 1:50,000 with a contour interval of 20 m. The purpose of DEM construction was to extract topographic parametres that may be associated with mosquito larval habitat formation, such as elevation, wetness index, flow distance to stream, aspect of land-surface and curvature. Elevation is directly related to temperature, which affects mosquito survivorship. Wetness index or topographic index represents land surface moisture content. It was calculated as ln(*A/TanB*) where *A *was the upslope contributing area and *TanB *was the local slope. Parametres *A *and *TanB *were derived using a multiple flow-direction algorithm [51]. Flow distance-to-stream may affect availability of the aquatic habitat and is calculated as the distance from a grid cell moving downstream to a stream grid cell defined by the Stream Raster grid [52]. The advantage of using flow distance-to-stream rather than simple distance-to-stream is that flow distance takes flow direction and landscape profile into consideration. Aspect of land surface is the terrain orientation and it ranges from 0 to 360 degrees. It is thus related to solar exposure, which may affect mosquito larval survivorship. Curvature is the measurement of the rate-change of the slope per unit distance [53], which may affect the stability of the aquatic habitat. The Terrain Analysis Using Digital Elevation Model (TauDEM) in ArcGIS was used to retrieve these parametres [52]. Due to its great range in values, the curvature was arctangent-transformed before conducting regression analysis. A three-dimensional model of the study area was constructed based on the DEM using ArcScene extension of ArcGIS [54].

Determination of an appropriate DEM scale is critical due to the effects of scale on the land-surface representation [55]. The DEM and topographic parametres were calculated for five different scales (20 – 60 m with an interval of 10 m), using the linear interpolation technique built in the ArcGIS spatial analyst module. To better differentiate presence and absence of habitat independent sample t-tests, were conducted to determine the most appropriate scale for each topographic parametre. In addition, seasonal consistency was considered so the models could be applicable to different seasons. The following were, consequently, chosen: wetness index at 30 m, aspect at 60 m, and curvature at 50 m, for both dry and rainy seasons. For flow distance, 20 m and 40 m were chosen for wet and dry seasons, respectively.

### Data analysis

#### Land-use and land-cover classification

In order to determine whether these three remote sensing tools are able to identify aquatic habitats and land-cover types that affect the survivorship of anopheline larvae [56], the remote sensing images were classified for land-cover types using a supervised classification method. A total of seven land-cover classes were used: farmland, pasture, natural swamp, forest, river/stream, road, and shrubs. Farmland was characterized either by the presence of an agricultural crop or bare ground that had been prepared for planting crops. Pasture was grassland-used for grazing or an area with a mixture of grass and shrubs. Natural swamp was characterized by the presence of emergent aquatic plants. Forest referred to areas with dense tree cover, normally with a closed canopy. Streams were classified as waterways less than one metre wide while rivers were more than one metre wide. Roads were surfaces reserved for motor vehicles and could either be tar-marked or not. Shrubs were short mature trees that were less than two metres tall. Each of the seven land-cover classes was determined based on their spectral signatures (expressed in terms of colour or greyness), texture (the smoothness of the object) and structure (the spatial arrangement). For example, healthy forest appears red on the image while the water is black dark in terms of reflectance under the combination of red, green, infrared bands. The main difference between forest and shrubs is in the spatial arrangement and smoothness of the crowns. Prior to classification being conducted, 10 random samples (plots) were visited for each of the seven land-cover classes in order to relate particular land-cover types to their specific spectral signatures, texture and structure. The images were classified and digitized using ArcView 3.3 [54].

#### Ground truthing of land-use and land-cover

Ground truthing was conducted in both dry and rainy seasons by direct field inspection of 185 points randomly selected by a script for random points generation in ArcView software. A misclassification matrix, which assesses the accuracy of land-use and land-cover classification, was calculated by comparing image classification and field observation results. Surface area of each land-cover type was estimated by first projecting the land-cover layer into WGS_84 UTM, Zone 36 N, and then using an Avenue script that calculates the surface area in ArcView.

#### Identification of aquatic habitats

Potential breeding habitats of anopheline mosquitoes in this study area included pools, ponds, trenches, abandoned gold mining sites, animal foot-prints, tyre tracks and others. Ten aquatic habitats for each habitat type were visited to ascertain their reflectance range, texture and structure on the imageries (aerial photos, Ikonos and Landsat 7). Identification of aquatic habitats was then performed based on the imageries. The classification accuracy was determined by overlaying all ground-identified and geo-referenced habitats in the dry season to the retrieved habitats from the imageries using ArcView. The number of correctly identified aquatic habitats was calculated for each land-cover type for the dry season only, because all the remote sensing images were taken during the dry season. The χ^2 ^tests were used to determine whether misclassification rates of aquatic habitats were significantly different among the three remote sensing types, whether some land-cover types had significantly more aquatic habitats and anopheline-positive habitats than expected based on the area size, and whether the proportion of anopheline-positive habitats differed among the land-cover types.

Aquatic habitats and anopheline-positive larval habitats are estimated based on parametres extracted from remote sensing images and DEM. A stepwise logistic regression analysis was used to determine important topographic features. The dependent variable was presence or absence of aquatic habitats. The independent variables were five topographic parametres derived from DEM: elevation, wetness index, flow distance to stream, aspect of land surface, and curvature. For this analysis, an equal number of "dummy" control sites were created in the study area that were not aquatic. The locations of these control sites were selected randomly, with the one criterion that they are at least 50 m from any observed aquatic habitat. This ensured that the control sites represented the same background environment as the observed aquatic habitats.

The probability of a grid cell suitable for aquatic habitat (*P*) was calculated by applying the resulting logistic model to the topographic parametres, using the relationship  where *f*(Topography, Landcover) refers to the final equation of the stepwise logistic regression on topographic parametres and land-use/land-cover variables. A map illustrating the predicted probability of aquatic habitat occurrence was generated using the map calculator function in the ArcGIS software. The study area was represented by a 4 km × 4 km rectangle or 133 rows by 133 columns, for a total of 17,689 grid cells. The misclassification rate of the model was calculated by comparing the observed habitat sites with those predicted by the model, using a cut-off probability of 0.5. That is, a site is considered to have an aquatic habitat if the predicted probability is ≥ 0.5, whereas aquatic habitats are considered absent if *P *< 0.5. This cut-off threshold probability gives an equal penalty for an actual habitat being classified as negative as for a negative site being classified as positive.

A stepwise logistic regression analysis was also used to determine whether the occurrence of anopheline positive larval habitats was associated with environmental variables. The dependent variable was the occurrence or absence of anopheline larvae in a habitat. The independent variables were the five topographic parametres obtained from DEM, the normalized difference vegetation index (NDVI), and the land-cover types. The NDVI value for each grid was obtained from the multispectral Ikonos image, using the standard method [50]. The probability of a grid cell containing anopheline-positive larval habitats was calculated using the formula described above. The misclassification rate of the model was calculated by comparing the observed habitat sites with a model prediction using a cut-off probability of 0.5.

## Results

### Identification of land-cover types

**Figure 1:**
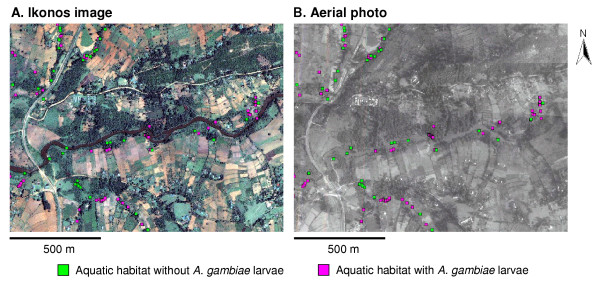
Example of Ikonos image (A) and panchromatic aerial photos (B) of the study area.

Supervised classification of land-cover types, based on the Ikonos image after ground truthing, revealed that farmland constituted 64.7% of the total area in the study area, while forest, pasture, shrubs and swamps represented 11.4%, 13.0%, 8.3%, and 0.8% of the land surface area, respectively (Table 1). Landsat TM7 identified farmland and river/streams with good accuracy (63.2–84.1%), but it showed low accuracy for the other land-cover types (0–23.1%). The Ikonos image provided a high degree of accuracy for identifying farmland, forest, pasture, river/streams, and road (72.7–96.8%), but it exhibited low accuracy for determining swamps and shrubs (33.3–56.3%). Overall, the misclassification rate of land-cover types in terms of area size was 10.8% based on Ikonos imagery, 22.6% for panchromatic aerial photos, and 39.2% for Landsat TM 7 imagery (Table 1). The Ikonos image provided more accurate identification of land-cover types than those based on panchromatic aerial photos or Landsat TM 7 images because the Ikonos image has a higher spatial resolution (1 metre) and is multispectral (Figure 1). Thus, the identification of the patchy and heterogeneous land-use types encountered in the western Kenya highlands can be better determined by Ikonos images.

**Table 1:** Area size of land-cover types and percentage of area size of each land-use and land-cover types being classified correctly using images from three remote sensors.

Land-cover	Area size (percentage)*	Aerial photos	Landsat TM 7	Ikonos
Farmland	8,733 (64.7)	88.9	84.1	96.8
Forest	1,533 (11.4)	76.9	23.1	92.3
Pasture	1,750 (13.0)	50.0	22.7	72.7
River/streams	136 (1.0)	70.6	63.2	86.4
Road	119 (0.9)	82.1	21.3	88.6
Shrubs	1,117 (8.3)	37.5	0.0	56.3
Swamp	105 (0.8)	33.3	0.0	33.3
Total	13,493 (100)	77.4	60.8	89.2

* Area size is in thousand square metres

### Distribution of aquatic habitats and anopheline-positive habitats among land-cover types

Ground survey found that the number of aquatic habitats increased 2.9 fold during the rainy season over the dry season (1,911 vs. 673). In both dry season and rainy season, river/streams and swamp had significantly more aquatic habitats than expected from the area size of each land-cover type in the study area, while farmland and forest had significantly fewer aquatic habitats (χ^2 ^= 4,418, df = 6; P < 0.0001 for the dry season; χ^2 ^= 2,455; df = 6; P < 0.0001 for the rainy season; Table 2). This was expected because depressions along river/streams and swamps facilitated stagnant water accumulation while farmland is mostly located on the hillside where water rarely stagnates. A total of 273 aquatic habitats (40.7%) were visually identifiable with the Ikonos image, 78 (11.6%) with the aerial photos, and 0 (0%) with the Landsat TM 7 image in the dry season. The combination of blue, green, and infrared on the multi-spectral Ikonos image facilitated the identification of water bodies even when water bodies were mostly covered by forest canopy.

**Table 2:** Distribution of aquatic habitats and anopheline-positive habitats in each land-use and land-cover type and percentage of aquatic habitats being correctly identified.

	Dry season		Rainy season		No. aquatic habitats correctly identified in the dry season		
	
Land-cover	No. habitat (percent)	No. anopheline-positive habitats (percent)	No. habitat (percent)	No. anopheline-positive habitats (percent)	Aerial photos (percent)	Landsat (percent)	Ikonos (percent)
Farmland	249 (37.0%)	123 (38.9%)	817 (42.7%)	402 (51.5%)	14 (5.6%)	0 (0%)	90 (36.3%)
Forest	55 (8.2%)	19 (6.0%)	107 (5.6%)	21 (2.7%)	2 (3.7%)	0 (0%)	24 (44.6%)
Pasture	87 (12.9%)	52 (16.5%)	481 (25.2%)	237 (30.4%)	20 (23.0%)	0 (0%)	45 (51.7%)
River/streams	133 (19.8%)	62 (19.6%)	87 (4.6%)	19 (2.4%)	26 (19.5%)	0 (0%)	53 (39.8%)
Road	3 (0.4%)	1 (0.3%)	147 (7.7%)	21 (2.7%)	0 (0%)	0 (0%)	0 (0%)
Shrubs	38 (5.6%)	16 (5.1%)	145 (7.6%)	30 (3.8%)	0 (0%)	0 (0%)	8 (21.1%)
Swamp	108 (16.0%)	43 (13.6%)	127 (6.6%)	50 (6.4%)	16 (14.8%)	0 (0%)	53 (49.1%)
Total	673 (100%)	316 (100%)	1,911 (100%)	780 (100%)	78 (11.6%)	0 (0%)	273 (40.7%)

A total of 316 and 780 anopheline-positive habitats were observed in the dry and rainy seasons, respectively. The distribution of anopheline-positive habitats among the seven land-cover types was proportional to the aquatic habitat distribution (χ^2 ^= 6.61, df = 6, P = 0.36). In the rainy season, however, significantly more anopheline-positive habitats were found on farmland and in pasture than expected based on the aquatic habitat distribution and significantly fewer anopheline-positive larval habitats were found in forest, river/stream, road, and shrubs (χ^2 ^= 82.21, df = 6, P < 0.0001; Table 2).

**Figure 2:**
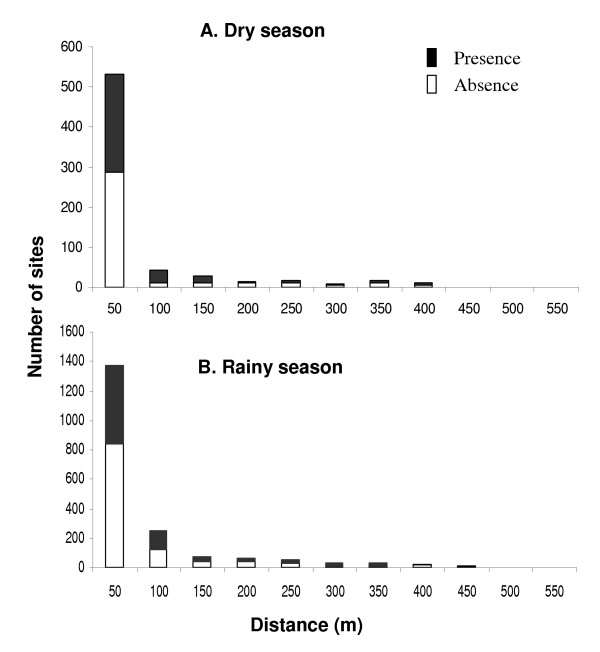
Distribution of anopheline larval habitats with respect to the distance to the nearest stream in the dry season (A) and rainy season (B).

**Figure 3:**
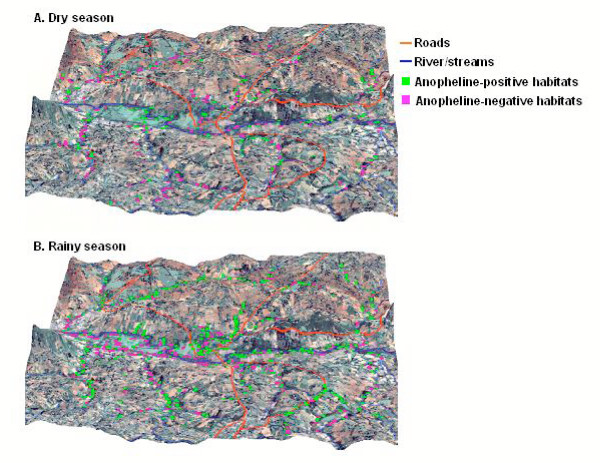
Three-dimensional map of the study area with all aquatic and anopheline-positive larval habitats overlaid in the dry season (A) and rainy season (B).

Anopheline larval habitats were generally clustered near the streams. For example, 78.1% and 68.3% of anopheline-positive habitats were located within 50 metres of streams in both the dry and rainy seasons (Figure 2). The average distance of anopheline larval habitats to the nearest stream in the dry season (mean = 44.3 m, standard error [SE] = 4.4) was significantly shorter than that in the rainy season (mean = 58.6 m, SE = 3.0, t = 2.53, df = 1098, *P *< 0.01). Anopheline-positive habitats were more concentrated in lower areas than in uphill areas. For example, 65.8% and 82.9% of anopheline-positive habitats were found on the valley bottom, between 1,400–1,440 metre elevation, during the rainy season and dry season, respectively. This became evident when the spatial distribution of the anopheline larval habitats was superimposed on the 3-dimensional image of the study area (Figure 3).

Among the 673 aquatic habitats observed in the dry season, 273 habitats (40.6%) were identified from the Ikonos image whereas aerial photos identified 10.6% and the Landsat TM 7 image did not identify any habitat (Table 3). The Ikonos image was better able to identify large habitats; for example, 70.9% of habitats larger than 100 m^2 ^were correctly identified, but only 22.2% of habitats less than 0.5 m^2 ^were correctly identified (Table 3).

**Table 3:** Distribution of larval habitat size in dry season and number of habitats correctly identified by three remote sensors.

Habitat surface area size (m^2^)	No. habitats (%)	Aerial photo (%)	Landsat (%)	Ikonos (%)
< 0.1	4 (0.6)	0 (0)	0 (0)	0 (0%)
0.1–0.5	45 (6.7)	0 (0)	0 (0)	10 (22.2)
0.5–1.5	104 (15.5)	11 (10.6)	0 (0)	34 (32.7)
1.5–5	174 (25.9)	18 (10.4)	0 (0)	55 (31.6)
5–10	105 (15.6)	18 (17.1)	0 (0)	45 (42.9)
10–100	186 (27.6)	19 (10.2)	0 (0)	90 (48.4)
>100	55 (8.2)	12 (21.8)	0 (0)	39 (70.9)
Total	673	78 (11.6)	0 (0)	273 (40.6)

### Computer-assisted identification of aquatic habitats and anopheline-positive habitats

**Figure 4:**
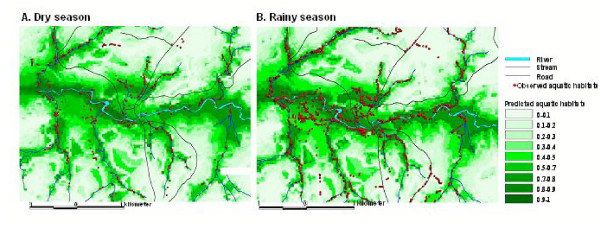
Predicted spatial distribution of aquatic habitats in the study area in the dry season (A) and rainy season (B).

Among the aquatic habitats, stepwise logistic regression analysis indicated that elevation, wetness, flow distance to river/streams, and curvature were significantly associated with the occurrence of aquatic habitats during the dry season (Table 4). Using the predicted probability of 0.5 as a cut-off point for misclassification, a total of 153 observed aquatic habitats were found to be misclassified, giving a misclassification rate of 22.7% (Figure 4). Similarly, during the rainy season, elevation, wetness, aspect, flow distance to river/streams, and curvature were significantly associated with the occurrence of aquatic habitats (Table 4). A total of 396 habitats out of 1,912 (20.3%) were misclassified in the rainy season (Figure 4).

**Table 4:** Stepwise logistic regression coefficients on the association between occurrence of aquatic habitats, occurrence of anopheline-positive habitats, and environmental variables.

Independent variable	Dependent Variable	Dry season	Rainy season
Occurrence of aquatic habitats	Elevation	-0.031	-0.042
	Wetness index	0.009	0.011
	Aspect	-	0.002
	Curvature	-0.290	-0.293
	Flow distance	-106.751	-30.004
	Constant	44.803	60.927
Occurrence of anopheline-positive habitats	Elevation	-0.568	-
	Flow distance	0.215	-
	Farmland	0.586	0.400
	Forest	-0.213	-0.978
	Pasture	0.747	0.403
	River/streams	0.391	-0.843
	Road	0.273	-1.360
	Shrub	0.317	-0.921
	Constant	-0.527	-0.432

Stepwise logistic regression analysis found that elevation showed a negative association, and flow distance a positive correlation with the occurrence of anopheline-positive habitats during the dry season (Table 4). Forest land-cover had a significantly negative association with the presence of anopheline-positive habitats while farmland and pasture showed a positive correlation. Using the predicted probability of 0.5 as a cut-off point for misclassification, 79 out of a total of 316 larval habitats (25.1%) were misclassified. During the rainy season, only land-cover type was significantly associated with the occurrence of anopheline-positive larval habitats (Table 4). In particular, the occurrence of anopheline-positive habitats was negatively associated with forest, river/streams, road, shrubs, but positively associated with farmland and pasture. A total of 141 habitats out of 780 (18.1%) were misclassified.

## Discussion

The aim of using remote sensing is to identify geographic features associated with mosquito breeding habitats, and ultimately to predict the spatial distribution of aquatic habitats, especially anopheline-positive habitats, for vector control programmes. Remote sensing images with different spatial and spectral resolutions are available. Among the satellite sensors used in this study, Landsat TM 7 has a spatial resolution of 30 × 30 metres and seven spectral bands (15 metres when using the panchromatic band), while Ikonos has a 1 × 1 metre sensor and four spectral bands. It was demonstrated that supervised classification based on Ikonos images had improved accuracy in determining land-use and land-cover types – a geographic feature significantly associated with the occurrence of anopheline larval habitats [47,57] – than aerial photos and Landsat TM 7 images. Eighty-nine percent of the area in this study site was correctly classified using Ikonos, while only 77% and 61% of the area was correctly identified by aerial photos and a Landsat TM 7 image, respectively. When aquatic habitats were visually identified using these three types of images, the Ikonos image had a high spatial and spectral resolution. About 41% of the aquatic habitats were identifiable based on the Ikonos image while only 11.6% were correctly identified using aerial photos and none were correctly identified using the Landsat TM 7 image. Landsat TM image under the 15 m panchromatic band could not be used for identification of aquatic habitats because most of habitats (81.8%) in this study site were less than 100 m^2 ^(Table 3), much less than the one-pixel size of Landsat image (225 m^2^). In principle, the detectable object has to be 1.5 the size of one pixel. Although more stable aquatic habitats are more productive to *An. gambiae *mosquitoes, *An. gambiae *larval habitats are generally smaller than the habitats identifiable by Landsat TM image (58, 59). These results suggest that the Ikonos sensor is superior to Landsat TM 7 image and aerial photos for determining potential anopheline larval habitats.

Several studies have used remote sensing to identify potential mosquito breeding habitats. Anyamba et al. [60] found that normalized vegetation difference index anomalies were associated with the availability of *Aedes *mosquito breeding habitats and Rift Valley Fever risks in Kenya. Sithiprasasna et al. [61] delineated stream networks from Ikonos satellite images and found that the risk of malaria infections was negatively correlated with distance-to-streams in Thailand. In Egypt, Hassan and Onsi [62] combined limited ground surveys with remote sensing techniques to identify mosquito breeding habitats in the Natroun Lakes area. In several past studies, vegetation cover, landscape structure, and distribution of water bodies were found to be associated with malaria risks [32]. In this study, it was shown that most aquatic habitats were close to streams and rivers (< 100 m). For example, 78.1% out of 314 and 68.3% out of 779 of anopheline-positive habitats were located within 50 metres of streams in the dry and rainy seasons respectively. The Ikonos image was able to identify 86.4% of the rivers and streams. Although Ikonos could only visually identify 40.7% of the aquatic habitats, the image and topographic derivatives could be used to improve the identification rates of aquatic habitats significantly. The computer model derived from topographic maps and remotely sensed parametres showed improved accuracy in determining the spatial distribution of aquatic habitats. The spatial distribution of more than 75% of aquatic habitats was predicted correctly. Whether these results are site-specific and whether they could be extrapolated to wide areas in the highlands is of interest.

Each remote sensor type has advantages in availability and utility for mosquito vector habitat determination. Aerial photos can be obtained for any area of interest, and they are generally not limited by cloud coverage because aerial photos can be taken when the sky is clear. Flying closer to the ground or using appropriate lens can increase the ground resolution of aerial photos. However, each photo covers a small area, image assembly based geo-referenced ground points is required to produce a mosaic of images for a large area, and thus may introduce geo-reference errors. In addition, aerial photos may suffer edge distortions caused by inappropriate camera position. Landsat TM 7 images are easily available at low cost in digital formats and a single scene covers a large area (swath width = 185 km), but the resolution is coarse. Ikonos images have the best spatial resolution for identification of major breeding habitats and human settlements, but they are more costly and often the availability of images is limited in areas where cloud coverage is significant and frequent. Each remote sensor type also had its limitations. The utility of panchromatic aerial photos is limited by the lack of infrared band capability, which is particularly useful for the identification of water bodies, especially when they are partially or completely covered by vegetation. While the Landsat TM 7 images have seven spectral bands, their 30 × 30 m ground resolution undermines their utility for identifying the patchy and discontinuous land-use characteristic of the western Kenya highlands. Although the Ikonos images have the advantage of high spatial and spectral resolution, this advantage is eroded when identifying land-cover types with similar spectral reflectance (e.g., fallow land vs. pasture, young forest vs. shrubs). Recognizing the advantages and disadvantages of these remote sensor types can help the selection of appropriate remote sensing images so that assist mosquito vector control efforts.

Successful prediction of the spatial distribution of anopheline mosquito habitats would allow vector control efforts to target the most productive larval habitats, resulting in a reduction of operational costs [25]. The statistical model used showed an 18–25% misclassification rate for anopheline-positive habitats. This classification error for anopheline-positive habitats could be due to inadequate understanding of factors regulating habitat productivity. Further knowledge of the underlying mechanisms of habitat productivity will help predict the spatial distribution of anopheline larvae in Africa.
